# The Trypanosome Flagellar Pocket Collar and Its Ring Forming Protein—*Tb*BILBO1

**DOI:** 10.3390/cells5010009

**Published:** 2016-03-02

**Authors:** Doranda Perdomo, Mélanie Bonhivers, Derrick R. Robinson

**Affiliations:** CNRS, Microbiology Fundamental and Pathogenicity, UMR 5234, F-33000 Bordeaux, France; doranda.perdomo@u-bordeaux.fr (D.P.); melanie.bonhivers@u-bordeaux.fr (M.B.)

**Keywords:** *Tb*BILBO1, flagellar pocket, flagellar pocket collar, cytoskeleton, flagellum, trypanosome, polymer, neglected tropical diseases

## Abstract

Sub-species of *Trypanosoma brucei* are the causal agents of human African sleeping sickness and Nagana in domesticated livestock. These pathogens have developed an organelle-like compartment called the flagellar pocket (FP). The FP carries out endo- and exocytosis and is the only structure this parasite has evolved to do so. The FP is essential for parasite viability, making it an interesting structure to evaluate as a drug target, especially since it has an indispensible cytoskeleton component called the flagellar pocket collar (FPC). The FPC is located at the neck of the FP where the flagellum exits the cell. The FPC has a complex architecture and division cycle, but little is known concerning its organization. Recent work has focused on understanding how the FP and the FPC are formed and as a result of these studies an important calcium-binding, polymer-forming protein named *Tb*BILBO1 was identified. Cellular biology analysis of *Tb*BILBO1 has demonstrated its uniqueness as a FPC component and until recently, it was unknown what structural role it played in forming the FPC. This review summarizes the recent data on the polymer forming properties of *Tb*BILBO1 and how these are correlated to the FP cytoskeleton.

## 1. Introduction

Kinetoplastids are protists that can be found in many environments and ecological niches. Some are free living, but others are parasitic and pose significant health burdens to human, animal and plant life in Africa, Asia and South America [1,2,3,4]. The genus *Trypanosoma* regroups the parasitic *Trypanosoma brucei rhodesiense* and *Trypanosoma brucei gambiense (T. brucei*), species that are zoonotic and anthroponotic, respectively. Together, they are the causative agents of Human African Trypanosomiasis (HAT or sleeping sickness) while *Trypanosoma brucei brucei* causes nagana in livestock [5,6]. The World Health Organisation (WHO) has estimated that up to 60 million people in Africa are exposed to sleeping sickness; however, the exact number of individuals and livestock infected is unknown, because many cases remain undiagnosed (http://www.who.int/en/).

A better understanding of the biochemistry, cell and molecular biology of these parasites should provide the identification of essential proteins and processes, which in turn may help in the development of new therapies against these targets. This is especially important since the current treatments include medications with toxic side effects but also there is considerable drug resistance. Further, there is little possibility of vaccine development because of a parasite based mechanism of thwarting the host immune system by constantly changing its surface coat using a mechanism called antigenic variation [7,8,9,10,11,12]. 

Trypanosomes possess certain single-copy organelles namely the nucleus, Golgi apparatus, mitochondrion, mitochondrial genome (kinetoplast), flagellum and the flagellar pocket (FP), which are potentially novel drug targets [13,14,15]. These organelles and their specialized compartments are of general scientific interest due to their multifunctional and complex roles. These structures can also have direct or indirect connections with the cytoskeleton of the cell [16,17,18,19,20,21,22,23] as observed with the microtubules, the main cytoskeletal polymers in trypanosomes. Microtubules are present in the flagellum, mitotic spindle and sub-pellicular corset; the latter is a complex array of microtubules that provide cell shape. Interestingly, trypanosome microtubules, and more specifically alpha and beta tubulin, can be post-translationally modified influencing their functions [24]. These sub-pellicular microtubules also have a posterior end plus polarity and are known to be required in cell growth, division and organelle segregation [23,25,26,27,28,29,30,31]. 

The cytoskeleton and the single copy organelles are subsequently intimately linked to the cycle and the life cycle of the parasite [26,29,31,32,33,34,35,36,37,38,39]. The FP and FPC are located in the posterior of the insect procyclic form and the mammalian bloodstream form cell, in a region of the pellicular membrane that is not underpinned by sub-pellicular microtubules. However, they are most likely attached to the corset and thus show some degree of interplay between these structures.

In this review, we focus on the flagellar pocket (FP), an essential endo-exocytosis compartment, and the cytoskeletal structure associated with it—the Flagellar Pocket Collar (FPC).

## 2. The Flagellar Pocket

A considerable amount of research has been focused on two life cycle stages of *T. brucei* because they can be cultured *in vitro* and be genetically manipulated. These are the procyclic form (PF) and the bloodstream form (BSF); the former is found in the insect vector and the latter in the mammalian host. Although each life cycle stage that is kept in the laboratory can vary from the naturally occurring strains, the tools developed for *T. brucei* study are based on these experimental cells.

**Figure 1:**
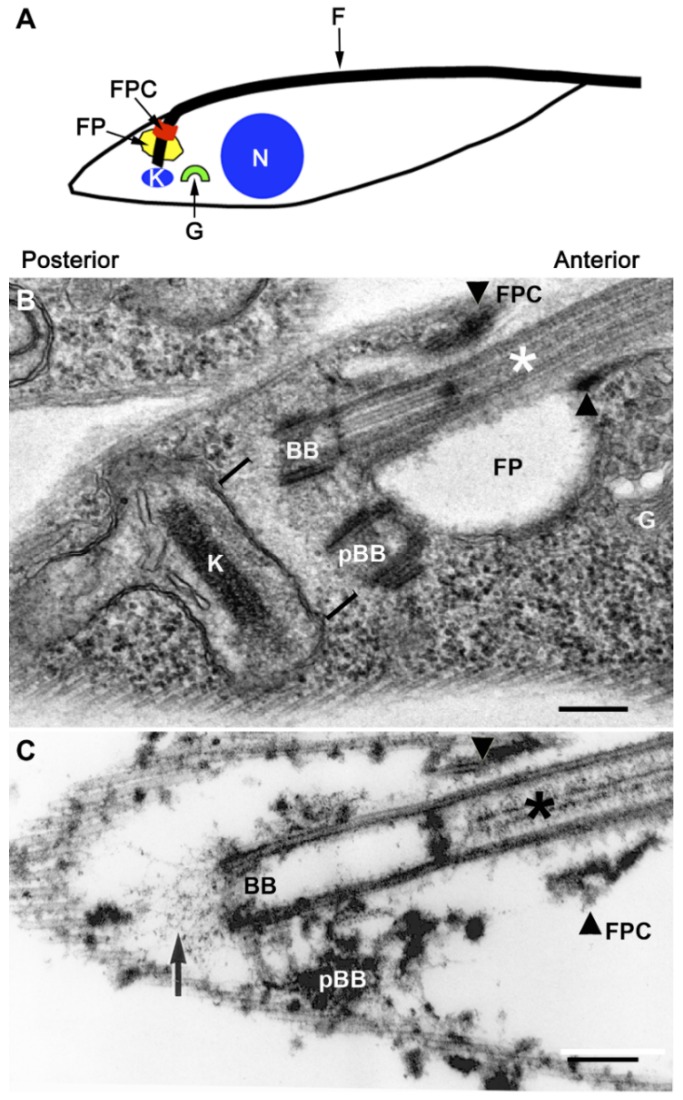
Schematic diagram of a *T. brucei* procyclic cell, showing cell polarity and organelle location. (**A**) The schematic shows the anterior and posterior regions of the cell. Note the polarization of organelles and structures to the cell posterior including the flagellar pocket (FP), flagellar pocket collar (FPC), Kinetoplast (K) and Golgi apparatus (G). (N), indicates the nucleus and (F) the flagellum. Figure 1A is reproduced from [44]; (**B**) Transmission electron micrograph of a thin section through the FP of a *T. brucei* procyclic cell. The asterisk indicates the axoneme. The mature basal body is denoted by **BB**. The immature basal body is denoted by **pBB**, and in this case it may have started maturing and has docked with the FP. The square brackets denote the ribosome free region. The kinetoplast (**K**) is the mitochondrial genome. The flagellar pocket is denoted by **FP** and the **FPC** is marked by arrowheads. The Golgi apparatus is denoted by **G**. The **FPC** forms the adherens-like junction containing BILBO1; (**C**) A thin section transmission electron micrograph similar to (**A**), but from a *T. brucei* procyclic “cytoskeleton”, where the soluble cell contents and membranes have been extracted with non-ionic detergent. The labels are the same as in (B) except the addition of the black arrow, which indicates the exclusion zone filaments of the tripartite attachment complex (TAC) [45]. Scale bars: 250 nm.

There is polarity in the *T. brucei* PF cell and this is orientated around the FP, which is located at the posterior of the fusiform cell, as is the mitochondrial genome (kinetoplast) and the Golgi apparatus (Figure 1A). The FP is the only site for exo-endocytosis and, as previously mentioned, is not underpinned by sub-pellicular microtubules [13,40,41].

The FP can be visualized using electron microscopy, as shown in Figure 1B. In Figure 1B, an electron micrograph thin section of a PF cell, we show the FP and related structures such as the flagellum, basal bodies, and kinetoplast. Serial block-face scanning electron microscopy has been used to measure FP volume and estimate it to be 1.5 × 10^−4^ μm^3^ in PF and 4.5 × 10^−4^ μm^3^ in BSF [42]. The FP lumen contains a carbohydrate-rich matrix, which, to date, has no clearly defined function [41] although it could theoretically function in some aspect of pathogenicity or defense, or alternatively it may serve in some mechanical manner to maintain FP integrity. It would be interesting to know the precise function of this matrix because it may be significant for parasite survival or pathogenicity. 

Important receptors are sequestered only into the FP, and elegant studies have shown that by forward swimming, BSF parasites can rapidly eliminate surface bound immunoglobulins and direct them into the FP where they are endocytosed and degraded [43]. Importantly, the major components of the endo-membrane system are located between the FP and the nucleus. This close organelle proximity is unlikely to be coincidental, and probably maintains high trafficking and recycling efficiency. For reviews on the subject, the reader is referred to [40,41].

## 3. The Flagellum and Its FP Associated Cytoskeleton

The flagellum originates from within the cell, but exits through the FP where it is attached to the cell surface. This lateral attachment along the cell body is mediated by a cytoskeletal structure called the flagellum attachment zone (FAZ) [31,39,46,47,48,49]. The flagellum continues along the cell to the anterior end where it eventually extends past the cell body [41,50]. Importantly, the flagellum is essential for cell motility and both PF and BSF cells swim predominantly with an anterior end forward motion [43,51,52,53,54]. The flagellum has numerous functions including motility, organelle segregation, attachment to insect host cells and regulation of cell length [23,55,56,57,58].

One of the first morphological features to be observed during the cell cycle is the transition through G1, and the nucleation of a subset of four microtubules that originate near the basal bodies, (please refer to “4MT” below), followed by the maturation of the immature basal body. Basal body growth is perfectly timed to mature during kinetoplast S phase and duplication [23,59,60,61,62]. At the end of the G1 cell cycle the immature basal body rotates 90° on its longitudinal axis and extends its distal end into the FP lumen to form a new flagellum [34,63]. The growing flagellum then physically attaches its distal end to the mature flagellum membrane forming a structure called the flagella connector (FC) that can be visualized by electron microscopy [37,64]. Interestingly, the FC has only been identified in PC. However, a structure termed “the groove” forms a discrete invagination of the cell body plasma membrane and is found in BSF at the distal tip of the growing new flagella, where it could function as the BSF equivalent to the FC of procyclic forms [65]. 

Later on in the cycle the cell will duplicate, which requires replication and segregation of all its organelles including the FP and the FP cytoskeleton. The latter includes a quartet of microtubules (4MT) also known as microtubule quartet (MTQ), which are considered to have the same polarity as the flagellar microtubules. However, no clear function has been attributed to these 4MT. Their microtubule-organizing centres (MTOC) are ill defined, but electron microscopy data indicate that they appear to be close to the basal bodies [46,66,67]. As the 4MT elongate, they will border and circumvent the FP and traverse a ring, or open ring/spring washer shaped, electron dense, structure at the neck of the FP, called the flagellar pocket collar (FPC) [34,44,68].

## 4. The Flagellar Pocket Collar

What is the FPC and why is it significant? The FPC forms a cytoskeletal boundary for the intersection of the pellicular and FP membranes as shown in Figure 1B (unpublished image). It is the structure that forms the distal region of the neck of the FP, which is also the site where the flagellum exits the FP [13,34]. Interestingly, the FPC remains attached to flagella after isolation using non-ionic detergent and high salt [68,69,70], suggesting that in order to isolate FPC proteins harsher treatments using chaotropic reagents need to be employed. In Figure 1C, we show a very similar image to Figure 1B, however, in this case, it is an image of a thin section through a non-ionic detergent extracted cell, known as a cytoskeleton, in which the cytoplasm and membranes have been extracted. The image illustrates the structures that are resistant to non-ionic detergent extraction including flagellum, basal bodies, FPC and the sub-pellicular microtubules. The latter are required to provide the fusiform shape of the cell. From Figure 1B,C, it is apparent that the FPC forms an adherens junction-like neck and this is proposed to maintain the pellicular, flagellar and FP membranes in close proximity. However, none of the genes coding for mammalian tight junction, adherens junction, or gap junction proteins have been identified in the trypanosome genome [71].

Gold particles up to 20 nm in diameter can enter the FP [67,72]. However, in BSF trypanosomes, when endocytosis is blocked by cold treatment, markers for endocytosis will accumulate at the membrane adjacent to the 4MT in a specialized channel within the pocket [67]. Additionally, elegant tomographical analysis of the *T. brucei* FP and associated cytoskeleton were also made by the Gull laboratory (University of Oxford, UK) [66] and in those studies they demonstrated that the daughter FP is formed by the growth and division of the mother FP. Both FP and FPC duplication is probably timed with new flagellum growth because they appear to rely on the new flagellum for physical segregation. This also indicates that there is a strict number control of single copy organelles since cell division in the absence of FP biogenesis, or an increase in FP copy number would most probably be lethal. Since the FP, FPC and flagellum are physically connected one obvious question to pose would be, is FP/FPC biogenesis possible in the absence of a flagellum?

This question was addressed using intraflagellar transport (IFT) mutants. IFT is a mechanism for flagella construction and is considered to be conserved in almost all eukarytotes (reviewed in [73]). The Bastin laboratory (Pasteur Institute, Paris, France) used PC trypanosome IFT knockdown mutants to specifically ask whether there are dependency relationships between the FP, FPC and flagellum. Through knocking down important IFT proteins and preventing flagellum formation they showed that a new FP and FPC can still be formed and segregated from the mother structures, but the FP is smaller and distorted and the FPC is less defined when probed with anti-*Tb*BILBO1 antibody [74], (*Tb*BILBO1 is a important protein of the FPC, and is described in detail below). After IFT knockdown, the FPC also appeared to be thinner when viewed by electron microscopy. This data indicates that there is no dependency relationship between the FP or the FPC that require the flagellum because both can be newly synthesized and localized to the expected position in the cell in the absence of a new flagellum [74]. 

## 5. BILBO1 and Flagellar Pocket Collar

**Figure 2:**
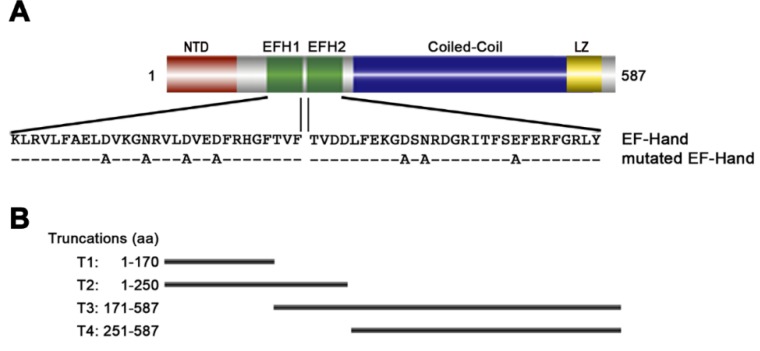
*Tb*BILBO1 domains: (**A**) The *Tb*BILBO1 protein consists of 587 amino acids, with a ubuquitin-like N-terminal domain (NTD, that ranges from amino acids 1–110), two EF-hand calcium-binding domains (EFH1 and EFH2, that ranges from amino acids 185–249), a long predicted CC domain and a leucine zipper (LZ, ranging from amino acids 533–578). The mutations in the EF-hand calcium binding domains are indicated below the wild-type amino acid sequence. Mutation studies have shown that the EF-hand domains modulate the morphology of polymers formed by *Tb*BILBO1; (**B**) The main truncations used to characterize polymer formation capacities are shown schematically below the secondary structure and labeled as truncation (T), from T1–T4.

In a search for essential minor cytoskeleton proteins as novel chemotherapeutic targets, we (the Robinson laboratory University of Bordeaux, France) identified an unusual protein of the FPC that was named *Tb*BILBO1 [44,68]. The primary and secondary amino acid sequence of full-length *Tb*BILBO1 (FL-*Tb*BILBO1) does not predict any localization or cytoskeletal functions. However, this protein has a N-terminus that contains an ubiquitin-like fold, followed by two EF-hand domains (amino acid residues ranging from 185–213 and 221–249), [75] which may be important in regulation, a large C-terminal coiled-coil domain (CC; amino acid residues ranging from 263-566) and a putative leucine zipper at its C-terminal (amino acid residues ranging from 534–578), suggesting possible roles in oligomerization and/or heterogeneous protein–protein interactions (Figure 2A).

A BLASTp analysis, using the National Center for Biotechnology (PubMed.gov) database has identified 20 orthologs of *Tb*BILBO1. This includes genes of non-mammalian species such as *Trypanosoma grayi* (parasite of crocodiles), the plant pathogen *Phytomonas serpens*, as well as the free living kinetoplastid *Bodo sultans* (*B. sultans*), (the *B. sultans* FP/FPC have not been extensively studied), suggesting that BILBO1 is essential and has been retained during speciation. Most of these genes have very similar locations in the genome with regard to their respective flanking genes, indicating that *Tb*BILBO1 gene synteny is preserved between these pathogens. Importantly, BLAST analysis has not identified *Tb*BILBO1 orthologs in any non-trypanosome genome. Further, *Tb*BILBO1 orthologs are present in all sequenced kinetoplastid genomes including *Leishmania* (a lethal parasitic protozoan that is present in many regions around the globe) and *Trypanosoma cruzi* (a lethal South American pathogen) [68,71,76]. However, since there is a large spectrum of life cycle modifications, host specificities and subsequent morphological phenotypes in these cells, the degree of variation in FPC structure is currently unknown. Notably, the presence of a *Tb*BILBO1 ortholog in *Bodo sultans*, a free-living species, has not been investigated and would be an interesting field to approach in order to characterize similarities and differences in BILBO1 orthologs and FPC structure and organization.

**Figure 3:**
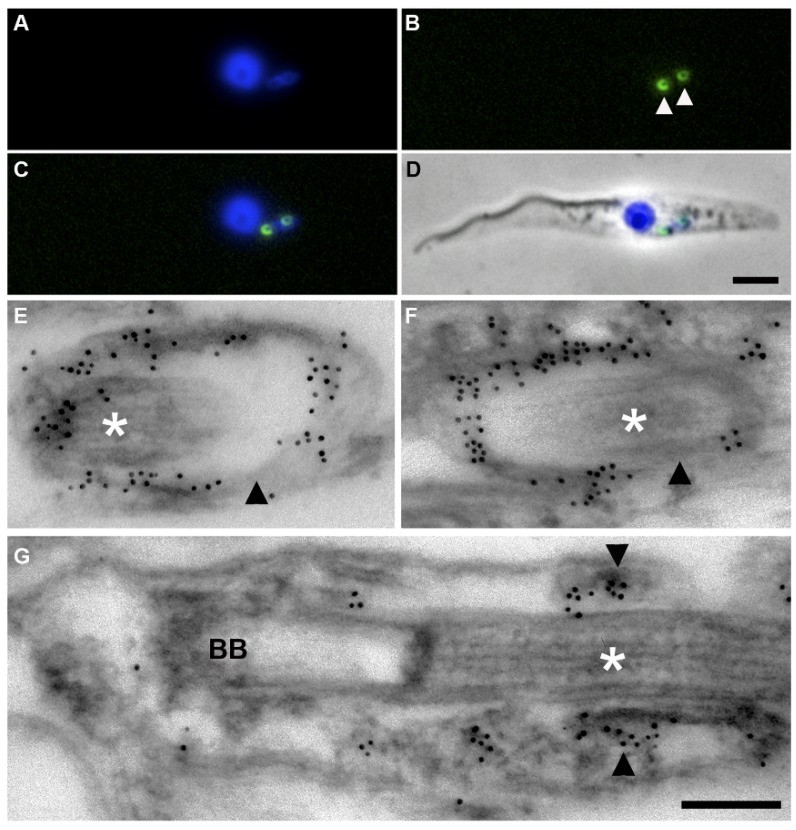
Cellular localization of *Tb*BILBO1 in procyclic cells: (**A**–**D**) Micrographs of a PF cytoskeleton labeled for immunofluorescence. The cell has duplicated its FPC and the daughter is located in the posterior rounded end of the cytoskeleton. The cytoskeleton was probed with monoclonal anti-*Tb*BILBO1 and DAPI. (**A**) DAPI; (**B**) monoclonal anti-*Tb*BILBO1 (white arrowheads); (**C**) merged DAPI, anti-*Tb*BILBO1; and (**D**) merged DAPI, anti-*Tb*BILBO1 and Phase contrast. Scale bar: 5 μm. (**E**–**G**) Electron micrographs of immunogold-labeled thin sections through the *T. brucei* FPC (black arrowheads). The FPC was labeled after detergent extraction using a monoclonal anti-*Tb*BILBO1 antibody followed by 10 nm gold-conjugated anti-mouse antibodies. Micrographs (**E**,**F**) are longitudinal sections of the FPC and (**G**) is a longitudinal section of the axoneme (BB, basal body). Asterisks indicate the axoneme traversing the FPC. Scale bar: 250 nm.

*Tb*BILBO1 can be visualized as a component of the FPC by immunofluorescence and electron microscopy using isolated flagella and cytoskeletons as shown by our laboratory, the Bastin Laboratory, He Laboratory (NUS Singapore), Dong, and Warren laboratories (Max F. Perutz Laboratories, Austria) [44,68,70,74,75,77]. Figure 3 illustrates immunofluorescence labeling of PF cytoskeletons using an anti-*Tb*BILBO1 monoclonal antibody made by our laboratory and demonstrate that the FPC forms a ring, or open ring-like/spring washer, structure that is located at the point where the flagellum exits the cell. In the example shown in Figure 3A–D, two FPC structures are observed and the newly formed daughter FPC is located in the rounded posterior of the cell. 

Immuno-electron microscopy (I-EM) of the FPC is shown in Figure 3E–F and illustrates that *Tb*BILBO1 is present on the full circumference of the FPC including the site of attachment to the axoneme as well as the region distal to the attachment site. I-EM of a longitudinal section of the flagellum and FPC demonstrates that *Tb*BILBO1 is present in the inner face of the FPC where it is directly opposite the flagellum (Figure 3G). Importantly, however, the proteins required for FPC-axoneme attachment are yet to be identified. 

More distal but closely associated to the FPC and possibly part of the same macromolecular structure, is the hook complex (also previously referred as the bilobe). Importantly, the hook has some morphological and physical overlap with the bilobe[16,77,78,79]. The main proteins so far identified in this “hook” are a membrane occupation and recognition nexus repeat protein (*Tb*MORN1), centrin and *Tb*LRRP1 [69,70]. The hook complex is the subject of another article within this set of reviews for the *Cells—Cilia and Flagella: Biogenesis and Function* Special Issue and thus will not be discussed here [80]. However, it is worth noting that RNAi knockdown of *Tb*LRRP1, an important hook protein, prevents FPC duplication. Nevertheless, no physical interaction between *Tb*BILBO1 and *Tb*LRRP1 has been documented [77].

## 6. BILBO1, the Cytoskeletal Ring of Power

Does *Tb*BILBO1 influence the FPC or other cytoskeletal structures? The famous J.R.R. Tolkien phrase *“One ring to rule them all”* answers this question in part and could easily be attributed to BILBO1 because knock-down of *Tb*BILBO1 by RNAi induced unusual morphological changes including cell arrest in a post-mitotic cell-cycle stage. Neither a new FP nor a FPC was formed after this, but many other unusual cellular phenomena were observed. When RNAi was induced in BSF cells, the FP and FPC were not formed, however big-eye like phenotypes were present[68], and electron microscopy analysis showed the accumulation of VSG-containing vesicles in the cytoplasm (data not shown). This would suggest a significant reduction of VSG recycling, and/or a bottleneck effect for vesicles containing VSG *en route* for the surface. 

In PF and BSF wild type cells the flagellum is attached to the cell via the basal body and the FAZ [46,50]. However, in both cell types after *Tb*BILBO1 RNAi knockdown, the new flagella were not attached along the cell body but merely connected to the cell via the basal body only. Additional studies on PF suggested that during knockdown the flagella were indeed never attached to the cell along its length [68]. Immunofluorescence using a FAZ monoclonal marker (L3B2) [46] demonstrated that new FAZ filaments were not formed in *Tb*BILBO1 RNAi-induced cells and the only FAZ signal observed remained associated with the maternal flagellum. This however does not preclude the possibility that any of the long list of FAZ proteins are indeed present in the FAZ zone [46,49,58,81,82,83,84]. Nevertheless, since cells lacking the FAZ structure always exhibit some degree of flagellum detachment, the presence of a functioning FAZ does seem unlikely [68]. It could also be argued that the absence of FAZ formation is because it requires flagella attachment early in flagellum biogenesis. However, the current data illustrate that flagellum formation and function are independent of FAZ formation and although detached, the new flagella were motile and relatively normal in terms of structure. Importantly, the FPC, FP or both were shown to be essential for FAZ formation [68]. 

These data suggest that the FPC or FP is directly or indirectly an organizing center for the biogenesis of numerous cytoskeletal structures such as the FC and the FAZ. Clearly, the interplay between the FPC and the FAZ is an important issue and also warrants further investigation.

*Tb*BILBO1 RNAi in PF cells induces elongation of the posterior ends. This indicates that flagellum attachment along the cell body is not required for flagella growth or movement between sub-pellicular microtubules. Since the FAZ is not detectable in knockdown cells, the new flagellum is either mechanically pushed to the cell posterior (possibly by hydrodynamic flow as the cell swims) using the unaffected mother flagellum [43], or the basal bodies are moved by the force of microtubule polymerization as the new subpellicular microtubules are being formed. A third option might be a combination of both mechanisms, or perhaps an alternative mechanism is responsible. Regardless of the machinery used, the associated elongated phenotype also raises the point that the FP or FPC may indirectly influence cell shape through cross talk with the sub-pellicular microtubule array. 

Gadelha and coworkers elegantly showed that the 4MT are associated with a membranous channel that connects the FP lumen to the extracellular space. When bloodstream cells were incubated with 5 nm gold particles and cooled to 0 °C they accumulated gold particles in this channel. The authors proposed that the channel could be a barrier, an active escalator, a free flow channel or a combination of the latter two, and is important for the movement of molecules into the pocket [67]. It is not known, however, if the channel is used for the secretion or egress of molecules or fluids. With respect to the FPC, it is unclear if the 4MT are present in the *Tb*BILBO1 RNAi phenotype since markers for them are limited, and attempts to visualize them by electron microscopy were inconclusive. In wild type cells, the 4MT pass through the FPC and then continue along the full length of the cell. If the 4MT are indeed implicated in forming the channel, it would not be unusual if they do indeed have additional functions and one obvious candidate is the delineation of the cleavage furrow site in cell division. 

Curiously, an additional microtubule, called the neck microtubule, is also observed paralleling the FAZ and on the opposite side of the 4MT. It is located at the FPC and terminates about 400 nm distal to the origin of the initiation of the paraflagellar rod (PFR), a crystalline flagellum structure that is required for flagellum motility and initiates where the flagellum exits the FPC [35,85]. Unfortunately, the function of the neck microtubule is also unknown, and its biogenesis and interaction with the FP or FPC needs to be investigated on a molecular level [66]. 

The importance of *Tb*BILBO1 has been demonstrated by RNAi, but other molecular methods have also been very informative about *Tb*BILBO1 function. For example, expression of FL-*Tb*BILBO1 or a *Tb*BILBO1 construct lacking the N-terminal domain (ΔNter-BILBO1) negatively affected *T. brucei* cell growth. Structural data regarding *Tb*BILBO1 has been difficult to obtain due to the intrinsic properties of the recombinant protein when expressed in a heterologous system (insoluble). Fortuitously, for our laboratory, the Dong laboratory purified and obtained NMR and crystal structures of the N-terminal domain of *Tb*BILBO1 (the region covering aa 1–110) revealing an unexpected ubiquitin-like fold with a conserved surface patch that would suggest a site of interaction with partner proteins [75,86]. They showed that expression *in vivo* of site-specific mutations within the patch or deletion of the N-terminal domain was lethal. Further, they demonstrated that the N-terminal domain was not needed for targeting *Tb*BILBO1 to the FPC *in vivo* but overexpression of ΔNter-BILBO1 induced growth inhibition, implying an important functional requirement for this domain and possibly inducing a dominant negative effect [75]. 

Additional and also very elegant studies by the Dong laboratory using untagged recombinant *Tb*BILBO1-EF hands (amino acid residues ranging from 178–250) reported that these domains bind calcium [87]. They also showed that filamentous assembly of *Tb*BILBO1 is mediated by the central coiled-coil domain and the C-terminal leucine zipper. Experiments using electron microscopy demonstrated that *Tb*BILBO1 assembles into anti-parallel dimers (head-to-tail) and that the leucine zipper at the C-terminus of the coiled coil (CC) domain of the anti-parallel dimers can bridge neighboring dimers forming an extended filament. 

From these data, it was thus proposed that *Tb*BILBO1 filaments could possibly associate via lateral and linear interactions between neighboring structures to form thick, long filaments *in vitro* and *in vivo* [87]. 

## 7. *Tb*BILBO1 Forms Polymers in Mammalian Cells

Taking from our studies on typanosome cytoskeletons and *in vitro* studies of the Dong laboratory, we theorized that the FPC probably consists of complex polymers. We therefore asked does BILBO1 influence FPC formation? To answer this question, the deconstruction of the above-mentioned *Tb*BILBO1 domains was carried out and tested by yeast two-hybrid (Y2H) and this provided valuable information regarding the *Tb*BILBO1-*Tb*BILBO1 association. The results showed that a positive interaction could be observed only when the CC domain was present on the *Tb*BILBO1 bait and prey proteins used in the Y2H assay [44].

The characterization of CC domains in several distinct proteins has shown that they are protein–protein interaction platforms capable of forming strong intermolecular interactions (based on electrostatic association) leading to specific oligomeric states and conformations [88]. We consequently developed the hypothesis that expression of *Tb*BILBO1 in a heterologous system would provide evidence for the formation of polymer structures. 

In this approach, *Tb*BILBO1 or truncated versions were expressed in mammalian cells (U-2 OS, these cells are classical adherent, model cells that were chosen for ease of growth, transfection and protein expression), (please refer to Figure 2B for truncations). Under these circumstances, *Tb*BILBO1 formed polymers that could be categorized by shape (complex or simple) and whether their termini were annular, comma or globular shaped. The results obtained demonstrated that the information for macromolecular assembly is encoded in the primary structure of the protein [44]. Although different polymers were formed, it was demonstrated using immunofluorescence that an increase of detected polymer fluorescence in U-2OS cells correlated to an increase in the number of complex termini (with a defined comma or globular end-form), relative to simple termini (straight end-form). This implies a concentration effect of *Tb*BILBO1 protein on termini. Polymers also increased in width over time implying a lateral *Tb*BILBO1-*Tb*BILBO1 interaction [44]. These data thus confirm the Dong laboratory results indicating that linear and lateral interactions can be formed by *Tb*BILBO1. 

Images obtained by electron microscopy using negative staining illustrated that the *Tb*BILBO1 fibers that formed in mammalian cells contained transversal striations with a mean periodicity of 46.9 nm thus indicating an organized and consistent formation process [44]. This highly ordered striation formation is not an effect of the U-2OS endogenous cytoskeleton machinery, because no ER, Golgi, or cytoskeleton structure was associated with, or affected by, *Tb*BILBO1 and thus demonstrating that it can indeed autonomously induce fiber formation [44]. Studies in the same heterologous system using truncated domains of *Tb*BILBO1 also demonstrated that only the CC domain is necessary for fiber formation. Interestingly, expression of the N-terminal domains with or without the EF-hands (domains are called T1, aa 1–170 or T2, aa 1–250), were either soluble or induced small aggregates, respectively but domains carrying the EF-hand and the CC domain formed linear polymers, and the CC domain alone formed spindle shaped polymers [44].

To evaluate the efficiency of polymer formation in a trypanosome environment, the same truncated forms of *Tb*BILBO1 that were used in U-2OS cells were expressed in *T. brucei* (Figure 2B). Interestingly, polymer formation was observed from domains carrying the EF-hands+CC (T3, the domain comprising of aa 171–587, which produced small fibers) or the CC domains alone (T4, aa 251–587 which produced elongated fibers). Neither the N-teminus (T1) nor with the addition of the EF-hand domains (called domain T2) had an involvement with polymer formation, and did not influence parasite growth. 

Surprisingly, expression of either domain T3 or T4 increased the endogenous expression of native *Tb*BILBO1 by over 4 fold, perhaps by sequestering exogenous *Tb*BILBO1 protein and forcing cells to increase expression. 

Following up on this work we also noticed that expression of native *Tb*BILBO1, T3 or T4 induced a detached flagellum phenotype in over 17% (for *Tb*BILBO1) and 35% (for T3 or T4) of the cells after 24 h induction. A detached new flagellum is a typical phenotype observed after induction of *Tb*BILBO1 RNAi and implies that FPC function is impaired. Again this maybe the result of native protein sequestration resulting in increased expression. The common domain present in full-length protein and the T3 and T4 forms is the CC region, thus implying that it is implicated in sequestration and the dominant negative effects. These results demonstrated that not only are the CC domains relevant for polymer formation, but expression induces a dominant negative effect that is eventually lethal for the parasite [44]. The results are also in accordance with the work of the Dong Laboratory who demonstrated that overexpression of the ΔNter-BILBO1 is lethal [75].

## 8. *Tb*BILBO1 Binds Calcium

Intra-filament (IF) structures presenting coiled-coil domains (as monomers or polymers) exhibit a structural shift towards bundles or networks in the presence of small cations such as Ca^+2^, Mg^+2^, Mn^+2^, Gd^+2^, due to the IF distinct surface charge patterns [89]. Considering this, the characterization and functional role of the EF-hand domains in *Tb*BILBO1 polymer formation was obviously a topic to pursue. 

The first *Tb*BILBO1 EF-hand is non-canonical (EF-1) and the second is canonical (Figure 2B). Using a purified recombinant *Tb*BILBO1 EF-hand domain protein (aa residues ranging from 177–250), and site-directed mutagenesis it was demonstrated that they are both able to bind two calcium ions, as measured by isothermal calorimetry (ITC), and that interestingly calcium binding induces a conformational change [44]. These observations encouraged the idea that there is specific structural change upon calcium presence in *Tb*BILBO1 *in vivo* and this may influence the binding or loss of binding of proteins. This would suggest that calcium binding could influence not only *Tb*BILBO1 polymer function but also its interaction with other proteins and perhaps its polymer forming properties.

**Figure 4:**
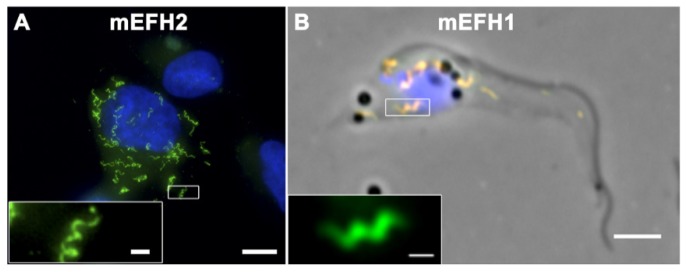
The effects of mutated *Tb*BILBO1 expression in a mammalian system and in *T. brucei*. (**A**) Immunofluorescence labeling of *Tb*BILBO1 carrying a mutation in EF hand 2 (mEFH2:myc) expressed for six hours in U-2 OS cells and probed using anti-NTD antibody. This mutated protein produces helical and ring shaped polymers. The inset shows a higher magnification of the boxed region. Scale bar: 10 μm; scale bar in magnified inset: 1 μm; (**B**) Immunofluorescence labeling of a *T. brucei* procyclic cytoskeleton expressing *Tb*BILBO1 carrying a mutation in EF hand 1 (mEFH1:myc). This mutation induces helical polymers. Cells were probed with anti-NTD antibody (green) and anti-myc (red) after 24 h of induction, and the images were merged. The inset shows the anti-NTD label alone. Scale bar: 5 μm; scale bar in magnified inset: 1 μm. Reproduced from [44].

In order to fully understand the role of the EF-hand domains in polymer formation, each EF-hand sequence was mutated (Figure 2A) and separately expressed using the U-2OS expression system (mEFH-1 or mEFH-2) or together (mEFH-1+2) in a *Tb*BILBO1 context. The results indicated that only the *Tb*BILBO1 carrying mEFH-2 was able to form polymers—helical, comma or annuli (Figure 4A). 

Surprisingly, the diameter of mEFH-2 formed structures (730 nm helix, 787 nm comma or 771 nm annuli) correlated well with the FPC diameter measured in *T. brucei* cells (842 nm). Curiously, when both (mEFH-1+2) are expressed in U-2OS cells only smaller polymers/aggregates are formed and these had no consistent shape. Even more striking, only 3.9% of cells are positive for such structures, suggesting a fast clearance of mEF-1+2 via the proteasome.

When mEFH-2 was expressed in mammalian cells it formed helices and annuli but it did not form these structures when expressed in trypanosomes. Oddly, it was mEFH-1 that formed polymers when expressed in trypanosomes and amazingly, these were visibly helical (Figure 4B) [44]. Importantly, these had a mean diameter of 747 nm, whilst the mean FPC diameter is 842 nm and would suggest that similarity of these dimensions is due to intrinsic *Tb*BILBO1 properties and not mere coincidence.

The difference in mEFH-1 or mEFH-2 capacities for polymer formation in the U-2OS cells compared to *T. brucei*, suggests the role of endogenous *Tb*BILBO1 (or other *T. brucei* proteins) in conjunction with mEFH-1, to originate helical polymers in the parasite. It also sheds light into differences within both EF-hand domains regarding their targeted specificity in the trypanosome cell compared to human U-2OS cells. 

Deletion or mutation of the EF hand domains when expressed for short time periods did not prevent the localization of the protein to the FPC. However, long expression of mEFH-1, mEFH-EF-2 or mEFH-1 (24 h) in PF *T. brucei* induced flagella detachment similar to phenotypes observed in *Tb*BILBO1 RNAi, (mEFH-1 = 32%, mEFH-2 = 11% and mEFH-1+2 = 25%). 

Finally, we wanted to test if we could prevent polymer formation in U-2OS cells by removing all available calcium. The addition of calcium chelator BAPTA-AM did not disrupt polymer formation, implying that once the polymers are constructed, they form a robust structure that is insensitive to calcium chelation [44]. 

## 9. *Tb*BILBO1 Calcium Binding and Polymer Shape

**Figure 5:**
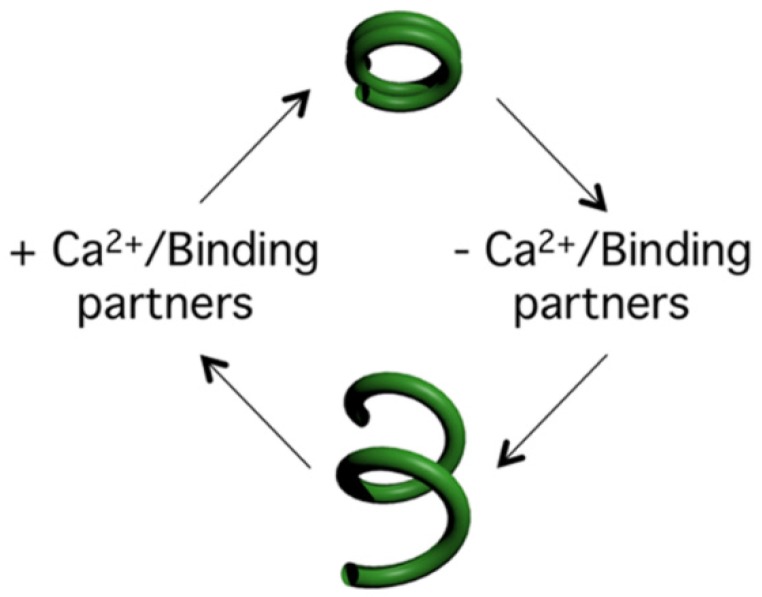
A simplified hypothesis for *Tb*BILBO1 polymer formation. Our current working hypothesis is that *Tb*BILBO1 has intrinsic polymer forming properties and these can be influenced to form annular or helical structures based on the tight binding of one or two calcium ions as well as parasite specific proteins. The balance between protein and calcium binding in turn will fluctuate based on the life cycle and cell cycle stage of the parasite.

Analysis of *Tb*BILBO1 demonstrates that it self-polymerizes, binds calcium, and plays a role in FPC formation and shape. It also exposes the functional characteristics of the EF-hand domains and how they modulate polymer form. Figure 5 provides a summary of the *Tb*BILBO1 polymer-forming hypothesis, which is that *Tb*BILBO1 has intrinsic polymer forming properties and these can be influenced to annular or helical structures based on the tight binding of one or two calcium ions, as well as parasite specific proteins. 

The fact that the EF-hand domains are not identical, the first one canonical and the second not, suggests a divergence in function and/or in complementary kinetics where one domain facilitates the other to bind calcium once itself has bound calcium. Of course, this difference could be due to other reasons such as the need for specific conformational changes and/or for binding to other FPC proteins, and this needs to be clarified.

While solving the crystal structure of full-length *Tb*BILBO1 would be most informative for understanding how the protein functions at the molecular level, this experiment is unlikely to work since BILBO1 forms an endless filament and multiple filaments tend to collapse into a condensed bundle [87]. An alternate way to solve this dilemma would be to combine the crystal structure of the BILBO1 coiled coil, which forms a stable antiparallel dimer [87], with that of the filament junction using truncated fragments of the protein. This combinatorial strategy would help reveal the structural details of full-length *Tb*BILBO1 and facilitate the analysis of key regions of the conformation of the protein, especially structural patches or domains that are accessible to therapeutic compounds. An effort to co-crystallize *Tb*BILBO1 and its potential partners, such as FPC5 [44], could also provide information into the biogenesis of the FPC in more detail. Since single or double mutation of key amino acids in the N-terminus of *Tb*BILBO1 leads to cell death when expressed *in vivo* it implies that indeed it could be a focal point of protein–protein interactions [75,86,87]. Furthermore, the localization of the leucine-zipper in the C-terminus, and the head-to-tail oligomer type polymers formed by *Tb*BILBO1, may function in a similar manner to the leucine-zipper peptide interactions found in the “belt and braces” model [90]. Thus, expressing *Tb*BILBO1 *in vivo* with mutated zippers would provide additional data on their function in polymer formation. 

## 10. Other Issues

*Tb*BILBO1 is not a particularly unusual protein, indeed, its domains are quite common, and are found in many other proteins and they have been well characterized. However, what is unusual is that these domains (a ubiquitin-like fold N-terminus, calcium binding regions, a coiled-coil domain and a leucine zipper) work in combination to confer the ability to form annular polymers. The Hook complex is physically very close to the FPC and is most likely associated with it, but BLAST and alignment analysis did not identify any significant similarities between *Tb*BILBO1 and a major protein of the hook complex, *Tb*MORN1. However, there are three small domains of homology between *Tb*BILBO1 and *Tb*LRRP1 (a hook protein) and they both share the amino acid sequence QHERT in their C-terminal domains. There are two small regions of homology between *Tb*BILBO1 and *Tb*Centrin 4 (hook protein) in the C-terminal domains of both proteins and also five small domains of homology between *Tb*BILBO1 and *Tb*Alpha tubulin. Although the FPC is probably linked to the FAZ via the 4MT only very minor similarities or regions of identity between *Tb*BILBO1 and FAZ proteins could be identified. Further, there is no sequence similarity between *Tb*BILBO1 and the unpublished partner protein we call FPC5 (a predicted kinesin with no known function) that binds to *Tb*BILBO1 [44]. The regions of similarity between *Tb*BILBO1 and other cytoskeleton proteins are often very small, thus the significance of these minor similarities remains unclear.

Apart from the 4MT and the FPC, the *T. brucei* FP has no other obvious cytoskeleton structure supporting its shape. The single *T. cruzi* ortholog *Tc*BILBO1 has 68% amino acid identity to *Tb*BILBO1, however, *T. cruzi* has a second endocytotic structure called the cytostome-cytopharynx and it has been shown, using serial electron tomography and scanning electron microscopy, that the cytostome-cytopharynx has a microtubule-based cytoskeleton [91,92,93,94,95]. Identification of the proteins involved in this cytoskeleton would be informative, as would their elimination, or the elimination of either the FP or cytostome-cytopharynx via CRISPR CAS9, for example [96,97]. It would also provide important data on the function of these organelles and whether either can compensate for the loss of function of the other.

Most quiescent and differentiated vertebrate cells have primary cilia and these are non-motile cilia that have axonemes made of nine peripheral doublet microtubules and no central pair (9+0) (for reviews see [98,99]). They function as sensory organelles, detect changes in fluid flow and initiate gene expression. When visualized by electron microscopy a small percentage of these cilia have, at their base, structural features that are similar to those of the trypanosome FP [100]. These features include the invagination of the pellicular membrane, coated pits and electron-dense material at the site where the cilia exits the cell, thus making a structural analog to the FP and FPC of trypanosomes. Importantly, however, *Tb*BILBO1 orthologs have not been identified in mammals. 

Primary cilia also have a pocket-like structure named the axonemal vesicle or sheath, which is thought to be Golgi-derived [101,102]. This vesicle presumably provides a compartment to allow intraflagellar transport for axonemal elongation within the cytoplasm. The mechanistic processes required for the biogenesis of ciliary pockets are unknown, and this presents novel challenges in understanding the mammalian ciliary pocket cytoskeleton or in making comparisons to a trypanosome FPC.

*Tb*BILBO1 has the intrinsic property to form circular or helical polymers. This property can be translated into the annulus formed by the FPC (Figure 5). Visualization of the FPC by immunofluorescence and I-EM raises the point that it is not always annular and is often visualized as horseshoe, or elliptical (unpublished data). One hypothesis derived from these data suggests that the FPC can grow, change shape and divide. Understanding if and how these shape changes are achieved is a major challenge and having investigated the FPC in PF cells it should not be taken for granted that the mechanisms needed for cytoskeleton function and form are the same in BSF parasites. This is especially important since it is known that kinetoplast segregation is different in BSF compared to PF, thus additional FPC based studies should now be done using BSF. 

Our current hypothesis for the function of *Tb*BILBO1 is that; due to its self-polymerizing properties it is a FPC scaffold protein forming a platform that supports other cytoskeleton proteins, which may maintain the FPC shape or initiate the site of duplication for other cytoskeletal structures such as the FAZ or the hook complex. Indeed we have identified at least one *Tb*BILBO1 partner protein (FPC5 [44] and unpublished data) supporting this hypothesis. 

*Tb*BILBO1 is indeed the “*One ring to rule them all”* because it has control over many cytoskeletal structures and the cell cycle, albeit directly or indirectly, and it is required to make many FP receptors invisible to the innate immune system. 

Our current challenges are to: (a) define if or what is the FPC association to its closest structural partner, the hook complex; and (b) identify products that interfere exclusively with the FP, FPC or hook complex. Such an approach will open novel avenues towards trypanosome disease prevention and chemotherapy. 

## Abbreviations

The following abbreviations are used in this manuscript:

HAT: Human Africa Trypanosomiasis
WHO: The World Health Organisation
FP: Flagellar Pocket
FPC: Flagellar Pocket Collar
PF: Procyclc Form
BSF: Bloodstream Form
4MT: Four microtubule quartet
MTQ: Four microtubule quartet
MTOC: microtubule organising centres
FAZ: Flagellum Attachment Zone
